# Improving AGB estimations by integrating tree height and crown radius from multisource remote sensing

**DOI:** 10.1371/journal.pone.0311642

**Published:** 2024-10-21

**Authors:** Xinyi Liu, Lili Dong, Shitong Li, Zhangmai Li, Yueyang Wang, Zhihui Mao, Lei Deng

**Affiliations:** 1 College of Resource Environment and Tourism, Capital Normal University, Beijing, China; 2 Forest Cultivation Department, Liaoning Provincial Academy of Forestry Sciences, Liaoning, China; 3 College of Emergency Management, Institute of Disaster Prevention, Hebei, China; 4 Resource and Environment Research Center, Chinese Academy of Fish Sciences, Beijing, China; Technical University in Zvolen, SLOVAKIA

## Abstract

Precise estimation of forest above ground biomass (AGB) is essential for assessing its ecological functions and determining forest carbon stocks. It is difficult to directly obtain diameter at breast height (DBH) based on remote sensing imagery. Therefore, it is crucial to accurately estimate the AGB with features extracted directly from RS. This paper demonstrates the feasibility of estimating AGB from crown radius (R) and tree height (H) features extracted from multi-source RS data. Accurate information on tree height (H), crown radius (R), and diameter at breast height (DBH) can be obtained through point clouds generated by airborne laser scanning (ALS) and terrestrial laser scanning (TLS), respectively. Nine allometric growth equations were used to fit coniferous forests (*Larix principis-rupprechtii*) and broadleaf forests (*Fraxinus chinensis* and *Sophora japonica*). The fitting performance of models constructed using only "H" or "R" was compared with that of models constructed using both combined. The results showed that the quadratic polynomial model constructed with "H+R" fitted the AGB estimation better in each vegetation type, especially in the scenario of mixed tall and short coniferous forests, in which the R^2^ and RMSE were 0.9282 and 25.30 kg (rRMSE 17.31%), respectively. Therefore, using high-resolution data to extract crown radius and tree height can achieve high-precision, global-scale estimation of forest above ground biomass.

## 1. Introduction

Forests are a vital component of the ecological environment. Accurately estimating their above ground biomass (AGB) is crucial for measuring their carbon sequestration and carbon emission capabilities, as well as for assessing their carbon budget [1]. Therefore, accurately estimating tree AGB is key to quantifying forest carbon sinks. Individual tree AGB within forests can be obtained through either direct measurement or indirect estimation methods [2]. Direct measurement is highly accurate, but time-consuming and costly [3, 4]. Remote sensing technology primarily facilitates indirect estimation of AGB [5]. Various types of satellite remote sensing data have proven effective in estimating the above ground biomass of extensive forests, including conventional optical imagery [6–8], waveform light detection and ranging (LiDAR) data [9, 10] and synthetic aperture radar (SAR) data [11, 12]. However, the accuracy of estimating forest tree AGB is low due to the limitation of spectral saturation or spatial resolution. In recent years, the utilization of Unmanned Aerial Vehicle (UAV) remote sensing for forest AGB estimation has surged, owing to its cost-effectiveness, adaptable takeoff and landing capabilities, safety features, ability to fly under cloud cover, and high spatial image resolution [13, 14]. It is also possible to install a LiDAR system on the UAV to gather point cloud data of dense forests through scanning, which can be utilized for estimating forest AGB [14–16].

AGB modeling is an indispensable component of remote sensing techniques for indirect estimation of forest AGB [17]. In recent decades, forestry scholars and researchers have investigated the AGB of numerous tree species and formulated various AGB models [18, 19]. The allometric growth equations used for AGB estimation are generally in the form of power functions, with most of them utilizing H and DBH as separate predictors or combining H and DBH as predictors [20, 21]. If allometric growth equations are established with only tree height as a predictor variable, this will inevitably bring some uncertainty to the estimation of forest tree AGB [22]. Izzati N A et al. (2023) demonstrated the importance of considering remotely sensed data in the estimation of AGB by analyzing the relationship between NDVI and above ground biomass [23]. VI (Vegetation Index) varies with the seasons, but the change in biomass of healthy vegetation within a year is negligible. This necessitates re-modeling, and when estimating biomass using different vegetation indices, uncertainty in its application may arise. Additionally, when combining different vegetation indices to estimate AGB, their application may introduce uncertainty [24]. When the canopy height is closed, the NDVI is affected by the "saturation effect", which may reduce the estimation accuracy [25]. The use of texture features in modeling to estimate above ground biomass of trees has been a topic of research [26], and Wu et al. demonstrated that the use of texture features in modeling can improve the accuracy of above ground biomass estimation of trees [27]. However, when modeling with texture features, resolution can affect the accuracy of AGB estimation. Different resolutions contain different texture information, which may lead to model instability. For structurally complex forests, obtaining accurate AGB solely through structural or spectral attributes is challenging. Goodman et al. emphasized the importance of canopy information in improving the accuracy of tree AGB estimation [28]. Lin et al. (2022) demonstrated the feasibility of the allometric growth equations constructed by H and R applied to the accurate estimation of forest tree AGB [29]. The relatively straightforward extraction of canopy information from UAV remote sensing data enables the integration of tree height with other canopy parameters to develop novel allometric growth equations [30]. The rise of UAV remote sensing technology has become a powerful tool for forest tree AGB estimation, and for high-resolution remote sensing imagery, the difficulty in obtaining the diameter at breast height has limited its application in AGB estimation. Therefore, it is urgent to explore the potential of using the canopy parameters obtained from high-resolution remote sensing images to establish a new anisotropic growth equation to obtain AGB.

In this study, we took coniferous and broadleaf forests as research objects, and used a combination of unmanned aerial data and ground-based data to study the following: the main contents include: 1)the use of crown radius derived from remote sensing images can effectively estimate AGB (Above ground biomass) and is more efficient compared to biomass estimation methods based on Diameter at Breast Height (DBH); 2) what combination of what kind of allometric growth model constructed with what combination of methods has the best effect in the estimation of AGB of individual trees.

## 2. Materials

### 2.1 Study area

**Fig 1:**
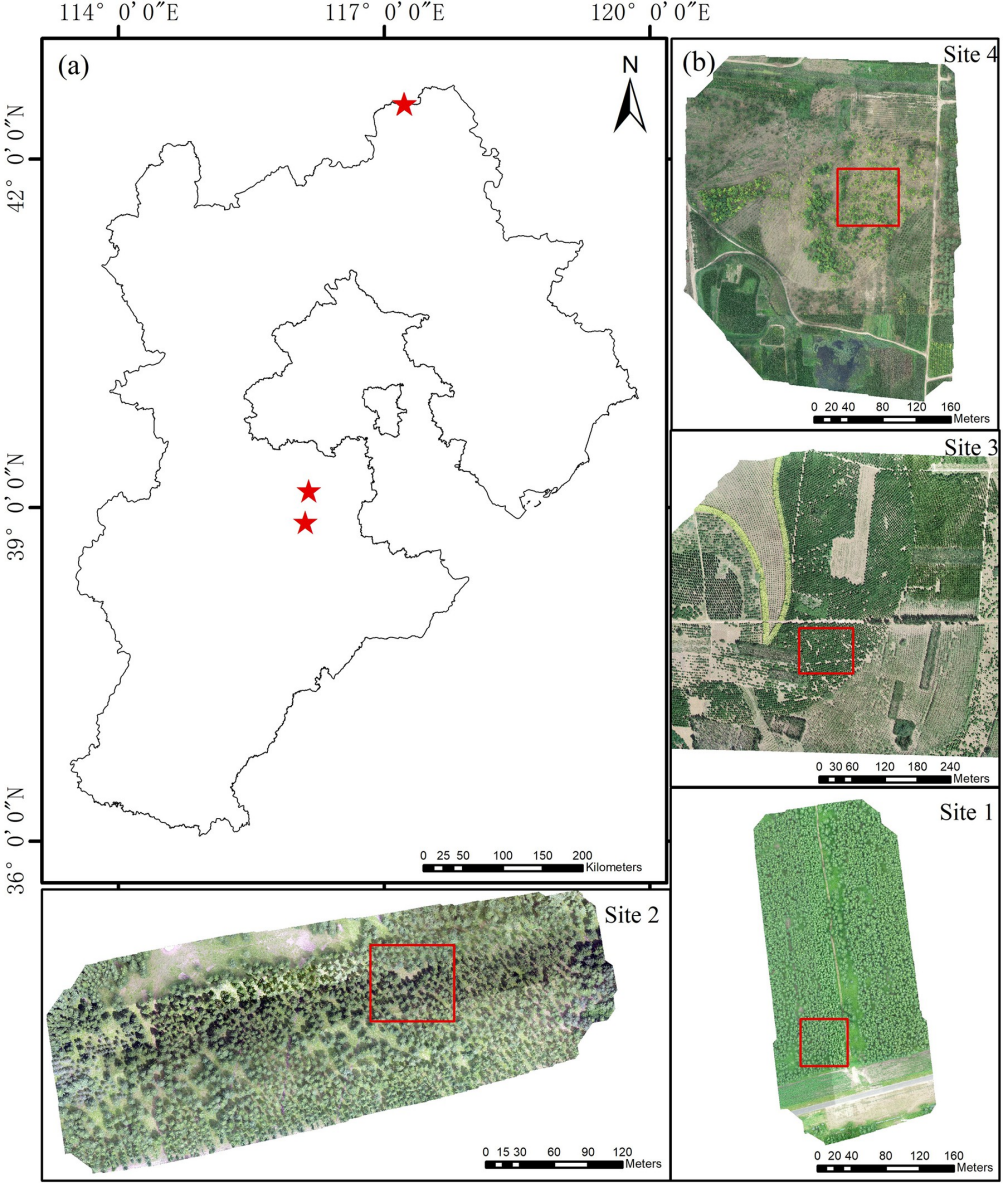
Study area. (a) Geographic location of the study area. (b) RGB images of 4 plots.

The study areas were all located in Hebei Province, China. One of the study areas is located in the Saihanba Mechanical Forest in Chengde City, with an altitude of 1100–1940 m. The region experiences a semi-arid and semi-humid cold-temperate continental monsoon climate. In the past five years, the average annual temperature is -1.4°C, the average annual precipitation is 450mm, and the annual sunshine duration is 2368h. The forested area reaches 76,600 hm^2^, with a forest coverage rate of 82%. The main tree species include deciduous *Larix principis-rupprechtii* (Fig 1).

The second study site is the Xiongan New Area, situated in the mid-northern latitude zone. It features a warm-temperate monsoon continental climate with four distinct seasons. In recent five years, the average annual temperature is 11.9°C, the annual precipitation is 495.1mm, and the annual sunshine duration is 2335.2h. The altitude elevation is 7–19 meters. The main tree species are *Fraxinus chinensis* and *Sophora japonica* (Fig 1).

### 2.2 Unmanned Aerial Vehicle (UAV) data acquisition and preprocessing

Data acquisition was performed utilizing a DJI M300 RTK, a multi-rotor drone outfitted with a DJI L1 camera. The DJI L1 sensor comprises both an RGB sensor and a LiDAR sensor, enabling simultaneous acquisition of RGB and LiDAR data. The L1 LiDAR boasts a ranging accuracy of 3cm@100m, facilitating precise localization. During the experiment, the UAV completed four flights, covering four distinct research locations, while flying at an altitude of 100 meters. The flights achieved an 80% overlap rate in both heading and side directions, and the point cloud density ranged from 581.938 to 1866.67 pts/m^2^. The parameters of the ALS laser scanning system are shown in Table 1.

**Table 1:** Parameters of the laser scanning system.

Technical Specifications	ALS	TLS
Maximum Distance Range	450 m	300 m
Positioning Accuracy	Horizontal: 10 mm+1 ppm	Horizontal: 8 mm+0.5 ppm
	Vertical: 15 mm+1 ppm	Vertical: 15 mm+0.5 ppm
Range Systematic Error	3 cm@100 m	4 mm@50 m
Laser Wavelength	905 nm	905 nm

The RGB and LiDAR data acquired were processed accordingly. The RGB data facilitated the generation of digital orthophotos (DOM) with resolutions of 2.0 cm and 2.7 cm for coniferous and broadleaf forests, respectively, aiding in visual interpretation. Processing of the ALS point cloud data included denoising, identification of ground points, normalization of the point cloud, segmentation of individual trees, and extraction of feature parameters for each tree [31, 32]. Initially, a smooth surface growth-based algorithm was utilized for detecting isolated points. Following that, an irregular triangular mesh progressive encryption filtering method was applied to filter the point cloud and conduct location identification, which led to the creation of a canopy height model (CHM). Using the CHM, normalized height was utilized to identify tall vegetation on the ground. Finally, the watershed point cloud segmentation method oriented to linear entity extraction is used to extract the parameter information of single wood. The specific parameter settings of point cloud processing are shown in Table 2.

**Table 2:** Parameter settings in TLS and ALS data processing.

Processing	Parameters	ALS	TLS
Ground point detection	Terrain inclination	60°	60°
	Iterative angle	6°	6°
	Iterative distance	0.6 m	0.6 m
Generation of CHM and high vegetation detection	Lower height value	2 m	0.2 m
	Higher height value	80 m	50 m
Individual tree trunk segmentation and feature parameter extraction	Average step length of trunk/canopy	2 m	0.15 m
	Growth step	1 m	0.5 m
	Minimum number of points contained in a single object	20	40

Individual tree height was determined by subtracting the highest Z-value recorded from the point cloud data from the height of the digital elevation model (DEM), while crown width parameters were extracted using the regional growth algorithm. The processes described above were executed using Point Cloud Automata (PCA) version 4.3.

### 2.3 TLS data acquisition and preprocessing

The TLS data was acquired utilizing a Stonex X300 laser scanner, a pulsed 3D laser scanner engineered for accurate measurements and rapid acquisition of large volumes of 3D point cloud data in complex environments. The measurement accuracy was 4mm@50m within a distance range of 300 m. TLS point cloud data is obtained in fine scan mode, and a single station is set up in various locations, resulting in an average point cloud density of 2281.92 pts/m^2^.

**Fig 2:**
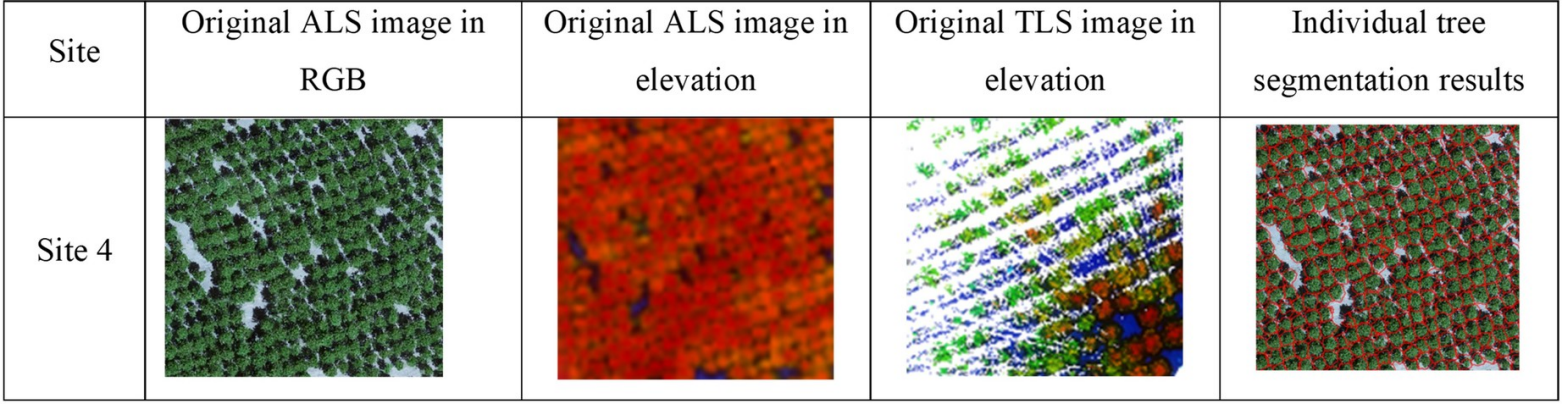
Segmentation results of individual trees based on point cloud data.

The primary processing steps for TLS are similar to those for ALS, encompassing location detection, point cloud normalization, detection of tall vegetation, segmentation of single-tree trunks, and extraction of feature parameters [31, 32]. The distance measurement function algorithm was employed to determine the DBH at a height of 1.3 meters above the ground [33–35]. Using Site 4 as an example, as shown in Fig 2, the individual tree segmentation is effective, with almost all the individual trees being accurately extracted. All of the afore mentioned procedures were carried out using Point Cloud Automata (PCA) v4.3.

Integration of ALS and TLS data. Leveraging the proximity of trunk positions extracted from both point cloud datasets, the fusion of point cloud data was achieved by correlating all other features in these two datasets based on their positional attributes [25, 36]. The same collection scheme was used for data acquisition in all study areas. The basic information of each tree species in the study area was obtained as shown in Table 3.

**Table 3:** Basic information of tree species in the study area.

Vegetation type	Tree species	Sample plot number	Tree height (m)	Diameter at breast height (cm)	Crown radius (m)	Number of trees
Coniferous Forest	*Larix principis-rupprechtii*	1	8.15~15.98	12.24~18.93	1.62~3.32	126
		2	15.11~22.11	13.88~35.46	1.98~4.97	127
Broadleaf Forest	*Fraxinus chinensis*	3	7.10~11.81	6.77~16.54	0.91~2.72	290
	*Sophora japonica*	4	2.03~5.69	10.75~18.11	0.64~2.89	82

As shown in Table 3, the height range of trees in sample plots 1 and 2 of the coniferous forest is 8.15~15.98m, 15.11~22.11m, and the crown radius range is 1.62~3.32m, 1.98~4.97m, respectively. The height range of trees in sample plots 3 and 4 of the broadleaf forest is 7.10~11.81m, 2.03~5.69m, and the crown radius range were 0.91~2.72m and 0.64~2.89m, respectively. Combined with the results of DOM visual interpretation, 126, 127, 290 and 82 trees were obtained from sample plots 1–4, respectively.

## 3. Methods

### 3.1 Allometric growth equation based on H and R

To estimate the above ground biomass of individual trees in forests by integrating H and R, it is essential to develop an allometric growth equation incorporating these two predictor variables. The tree height and crown radius were combined in the form of exponential, power function, linear, logarithmic and second-order polynomials to construct a new allometric growth model, and a total of nine models were established, as shown in Table 4.The most appropriate allometric growth equations based on H and R were compared and analyzed by trying different combinations of forms.

**Table 4:** Forms of allometric growth models.

Model Type	Model form	Model code
Power function	$W=aH^{b}$	Eq.1
Power function	$W={a(R)}^{b}$	Eq.2
Power function	$W={a(H+R^{2})}^{b}$	Eq.3
Power function	$W={a(HR)}^{b}$	Eq.4
Exponent	$W=ae^{b(H+R)}$	Eq.5
Linear	W=a(H+R)+b	Eq.6
Logarithm	W=aln(H+R)+b	Eq.7
Power function	$W={a(H+R)}^{b}$	Eq.8
Quadratic polynomial	$W={a(H+R)}^{2}+b(H+R)+c$	Eq.9

In Table 4, W represents individual tree above ground biomass (AGB) in kilograms (kg), H represents tree height in meters (m), R represents crown radius in meters (m), and a, b, and c are coefficients of the allometric growth model.

In order to determine the coefficient values in each allometric growth model, the parameters of single-tree H and DBH obtained in Section 2.3 were used to fit the isokinetic growth model based on the H and DBH of *Larix principis-rupprechtii* and ash and acacia in the Forestry Carbon Sink Measurement and Detection Technical Procedure [37]. The corresponding allometric growth models for tree species in the protocol are shown in Table 5.

**Table 5:** Allometric growth models of different tree species.

Vegetation type	Model form	Model code
*Larix principis-rupprechtii*	$W=0.1179{\left({D_{DBH}}^{2}H\right)}^{0.815}$	Eq.10
*Fraxinus chinensis* and *Sophora japonica*	$W=0.093{\left({D_{DBH}}^{2}H\right)}^{0.869}$	Eq.11

In Table 5, W represents individual tree above ground biomass (AGB) in kilograms (kg), D_DBH_ represents individual tree diameter at breast height in centimeters (cm), and H represents tree height in meters (m).

### 3.2 Scenario setting

Nine allometric growth models were fitted for *Larix principis-rupprechtii*, *Fraxinus chinensis* and *Sophora japonica*. To further investigate the applicability of the models across different tree species, comparative analyses of the models were conducted for combinations of *Fraxinus chinensis* and *Sophora japonica*, as well as *Larix principis-rupprechtii* with *Fraxinus chinensis* and *Sophora japonica*. By comparing the fitting performance of each model in the combinations of *Fraxinus chinensis* and *Sophora japonica*, and *Larix principis-rupprechtii* with *Fraxinus chinensis* and *Sophora japonica*, the most suitable allometric growth model for specific tree species combinations was determined. Various combinations of tree species are shown in Table 6.

**Table 6:** Combination forms among tree species.

Scenario	Tree type	Plot combination situation	Explanation
Scenario 1	*Larix principis-rupprechtii*	Plot 1	Shorter coniferous forests
Scenario 2	*Larix principis-rupprechtii*	Plot 2	Taller coniferous forest
Scenario 3	*Larix principis-rupprechtii*	Plot 1 + Plot 2	Mixed tall and short coniferous forest
Scenario 4	*Fraxinus chinensis*	Plot 3	Taller broadleaf forest
Scenario 5	*Sophora japonica*	Plot 4	Shorter broadleaf forest
Scenario 6	*Fraxinus chinensis*, *Sophora japonica*	Plot 3 + Plot 4	Mixed tall and short broadleaf forest
Scenario 7	*Larix principis-rupprechtii*, *Fraxinus chinensis*, *Sophora japonica*	Plot 1 + Plot 2 + Plot 3 + Plot 4	Mixed tall and short coniferous-broadleaf mixed forest

### 3.3 Validation

For the coniferous forest (*Larix principis-rupprechtii*) and broadleaf forest (*Fraxinus chinensis* and *Sophora japonica*), two study areas were selected separately for experiments. 70% of the data was used for model construction (obtaining model coefficients), and 30% was used for accuracy validation. The performance of each model was evaluated using metrics like the coefficient of determination (R^2^), root mean square error (RMSE), and relative root mean square error (rRMSE). The formulas are as follows:

$$R^{2}=1-\frac{\sum_{i=1}^{n}{\left(y_{i}-{\hat{y}}_{i}\right)}^{2}}{\sum_{i=1}^{n}{\left(y_{i}-\bar{y}\right)}^{2}}$$ (1)

$$RMSE=\sqrt{\frac{\sum_{i=1}^{n}{\left(y_{i}-{\hat{y}}_{i}\right)}^{2}}{n}}$$ (2)

$$rRMSE=\frac{RMSE}{\bar{y}}\times 100\%$$ (3)

where n is the tree used to evaluate the model, *y*_*i*_ and ${\hat{y}}_{i}$ represent the reference and predicted values, respectively, and $\bar{y}$ is the mean of the reference values.

## 4. Results

### 4.1 Estimation of AGB using different allometric growth equations

**Fig 3:**
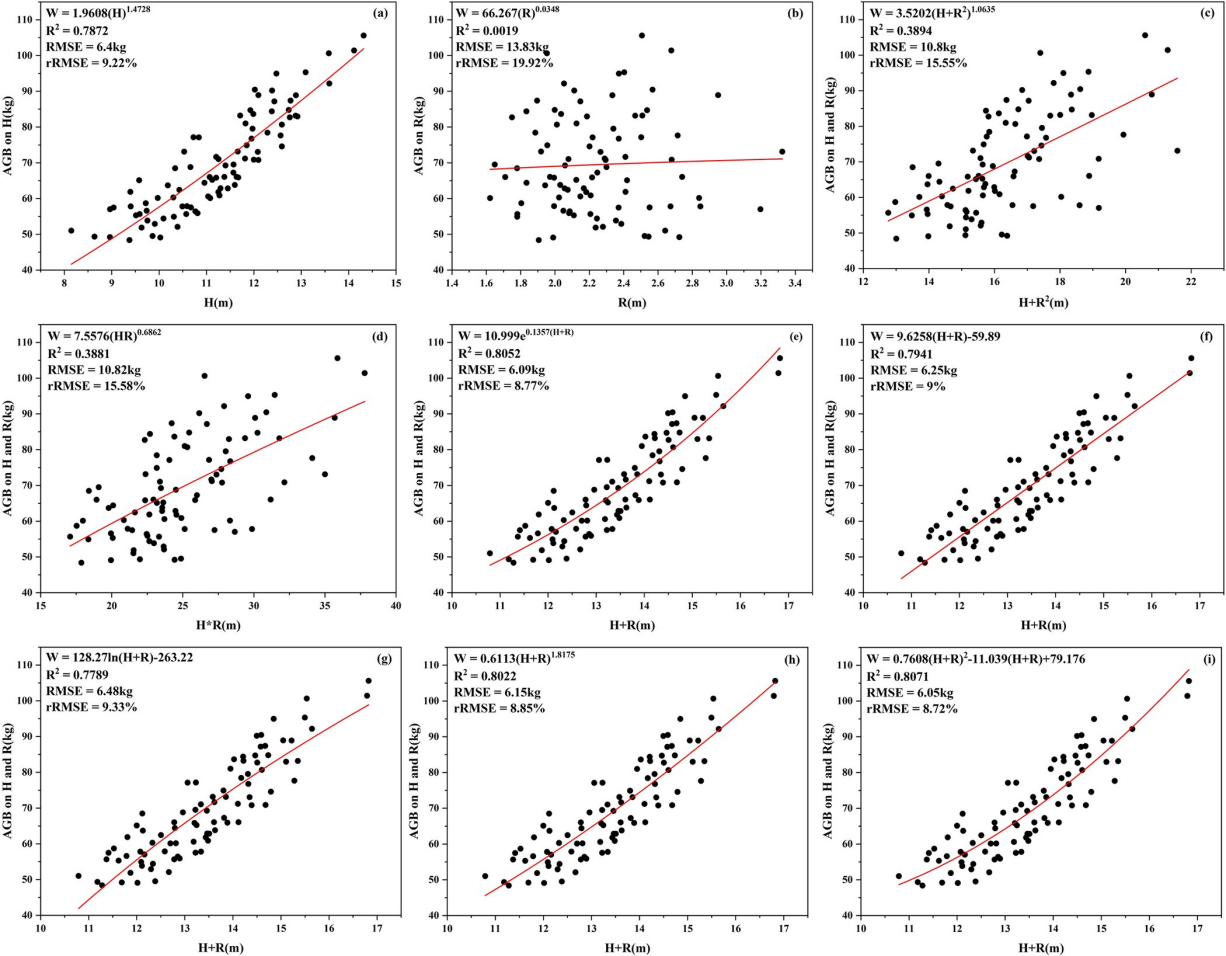
Fitting graph of 9 models for Scenario 1.

Based on the content of section 3.1, nine allometric growth equations were fitted to the vegetation type characterized by shorter coniferous forests (Scenario 1). The fitting performance of these nine models is illustrated in Fig 3.

The allometric growth model constructed with both H and R for AGB estimation in Scenario 1 outperforms the models constructed with only "H" or "R" (Eq.1 or Eq.2), and the quadratic polynomial model constructed with "H+R" (Eq.9) achieves the highest R^2^. Specifically, the best fitting model is the Eq.9 model, with R^2^ and RMSE of 0.8071 and 6.05 kg (rRMSE 8.72%), respectively. The models constructed only with "H" or "R" (Eq.1-2) have lower R^2^ compared to the Eq.9 model, especially Eq.2, with an R^2^ of only 0.0019. Apart from the Eq.9 model, three other models (Eq.5-6 and Eq.8) also exhibit higher R^2^ than the Eq.1-2 models.

### 4.2 Estimation of AGB in different scenarios

**Fig 4:**
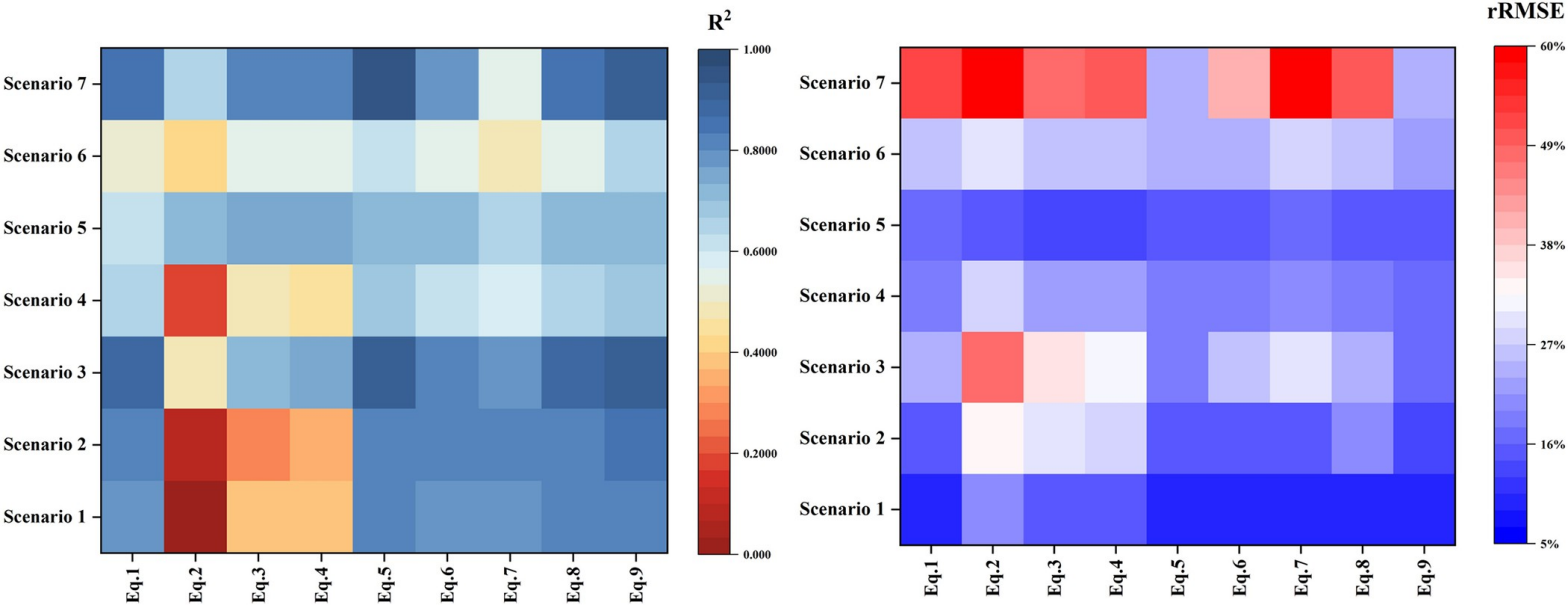
Accuracy graph of 9 models constructed for 7 Scenarios.

In addition to fitting Scenario 1 with a combination of H and R to construct models in each functional form in section 4.1, six other scenarios were designed in 3.2 with vegetation types covering diverse tree populations to analyze the applicability and performance of the models under different combinations of tree species by comprehensively evaluating the model accuracies. The accuracy of fitting each of the seven scenarios using the nine models is shown in Fig 4.

The allometric growth models constructed with both H and R for AGB estimation in the three scenarios of coniferous forests (Scenarios 1–3) outperform the models constructed with only "H" or "R" (Eq.1 or Eq.2). Additionally, the quadratic polynomial model constructed with "H+R" (Eq.9) consistently achieves the highest R^2^. Specifically, in Scenarios 1–3, the R^2^ of the Eq.9 model is above 0.8, while the R^2^ of the Eq.1-2 models are significantly lower, with Eq.2 model having an R^2^ of less than 0.5. In the taller coniferous forest (Scenario 2), besides the Eq.9 model, four other models (Eq.5-7 and Eq.8) exhibit higher R^2^ compared to the Eq.1-2 models. In the mixed tall and short coniferous forest (Scenario 3), apart from the Eq.9 model, the R^2^ of the Eq.5 model is higher than that of the Eq.1-2 models.

In the broadleaf forest (Scenarios 4–6), the estimation performance of models constructed with both H and R is superior to that of models constructed with only "H" or "R" (Eq.1 or Eq.2), and the quadratic polynomial model constructed with "H+R" (Eq.9) consistently achieves high R^2^ values. Specifically, the R^2^ values of the Eq.1-2 models are significantly lower than those of the Eq.9 model, with Eq.2 model having an R^2^ as low as 0.1975 in the taller broadleaf forest (Scenario 4). In the shorter broadleaf forest (Scenario 5), the best fitting model is the power function model constructed with "H+R^2^" (Eq.3), with an R^2^ of 0.7466 and RMSE of 4.11 kg (rRMSE 13.71%). Compared to the optimal model (Eq.9) in Scenario 4, the RMSE of the Eq.9 model in Scenario 5 (4.26 kg) is only 0.15 kg larger than that of the Eq.2 model, and the rRMSE (14.24%) is only 0.53% larger than that of the Eq.2 model. Hence, the quadratic polynomial model developed with "H+R" (Eq.9) can be consistently applied to estimate AGB in broadleaf forests. In Scenario 4, besides the Eq.9 model, two other models (Eq.5 and Eq.8) exhibit higher R^2^ values than models constructed with only "H" or "R" (Eq.1 or Eq.2). In the mixed short and tall broadleaf forests (Scenarios 5–6), besides the Eq.9 model, five other models (Eq.3-6 and Eq.8) exhibit higher R^2^ values than the Eq.1-2 models.

In the mixed coniferous and broadleaf forest (Scenario 7), the estimation performance of models constructed with both H and R is superior to that of models constructed with only "H" or "R" (Eq.1 or Eq.2), and the quadratic polynomial model constructed with "H+R" (Eq.9) exhibits relatively high R^2^ values. Specifically, the RMSE of the Eq.9 model is 20.9 kg (rRMSE 24.13%), while the relative RMSE of the Eq.1-2 models is greater than 40 kg, with values of 45 kg (rRMSE 51.95%) and 51.21 kg (59.12%), respectively. The best fitting model is the power function model constructed with "H+R" (Eq.5), with an R^2^ of 0.9396 and RMSE of 20.62 kg (rRMSE 23.81%). The next best model is the Eq.9 model, with an RMSE (20.9 kg) only 0.28 kg larger than that of the Eq.5 model, and an rRMSE (24.13%) only 0.32% larger than that of the Eq.5 model. Therefore, the quadratic polynomial allometric growth equation constructed with "H+R" (Eq.9) can be uniformly used to estimate AGB of single trees in the structure of mixed coniferous and broadleaved forests. Besides the Eq.9 model, two other models (Eq.5 and Eq.8) exhibit higher R^2^ values than the Eq.1-2 models.

## 5. Discussion

### 5.1 Crown radius as an efficient AGB predictor

Using high-resolution remote sensing data, especially based on unmanned aerial vehicles and LiDAR data, is crucial for obtaining accurate crown information to precisely estimate single-tree above ground biomass. Allometric growth equations, which rely on tree height and crown diameter, are crucial for precise estimation of single-tree AGB, thereby making significant contributions to improving forest management practices [38]. For the seven scenarios, constructing allometric growth models solely based on "R" as the predictor variable resulted in relatively low R^2^ values. Specifically, in the taller, shorter, and mixed tall and short coniferous s, as well as in the taller and mixed tall and short broadleaf forests (Scenarios 1–4 and 6), the R^2^ values were less than 0.5. However, as indicated in Section 4, combining H with R to construct allometric growth models yielded better fitting results. Potential reasons for this phenomenon include: while different tree species may exhibit similar tree heights, there can be considerable variations in diameter at breast height (DBH), resulting in substantial errors if only tree height is considered. Dey & Islam (2021) explored the relationship between H and DBH, further supporting the correlation between DBH and H [39]. Additionally, according to Attocchi et al. (2015), the crown radius of trees is influenced to some extent by DBH [40]. Brūmelis et al. (2020) also demonstrated the existence of an allometric growth relationship between crown area and DBH, implying the correlation between DBH and R [41]. Therefore, DBH, tree height, and crown radius are all strongly correlated, and incorporating R as an additional dimension essentially enhances the accuracy of tree type determination. This may be one of the reasons why adding R leads to more accurate results.

Lin et al. (2022) proposed the concept of using an "equivalent circle" to calculate crown radius, whereby the radius corresponding to the crown area is computed based on the area of a circle with equivalent area as the crown area [29]. This calculated radius is utilized as the crown radius for AGB estimation. However, due to the irregular crown shapes of some trees in the study area, the "equivalent circle" did not adequately cover these trees. Furthermore, the study focused solely on a single tree species, making it challenging to apply to large-scale tree AGB estimation. In contrast, our study utilized nine models to fit different coniferous forests of varying heights, broadleaf forest, and mixed coniferous and broadleaf forests separately, selecting models with stronger applicability for global-scale forest tree AGB estimation. Furthermore, in global-scale forest AGB estimation using satellite remote sensing data, the importance of crown information may be emphasized because of the difficulties in obtaining diameter at breast height (DBH) data, while acquiring crown width parameters is comparatively more achievable [42, 43]. The study revealed a strong correlation between crown width data and single-tree AGB, indicating promising opportunities for leveraging satellite remote sensing technology to estimate forest tree AGB in the coming years [28].

The inclusion of crown radius (R) can be applied for accurate estimation of plot-level AGB. Since various methods, such as drones, cannot provide diameter at breast height (DBH), it is challenging to apply commonly used allometric growth equations in forestry. However, remote sensing methods can easily obtain tree height (H) and crown radius (R), thus combining them can greatly save manpower, resources, and time. Additionally, there are methods utilizing LiDAR point cloud segmentation to obtain R and H for estimating DBH [44]. However, firstly, the accuracy of single-tree segmentation results from LiDAR point cloud segmentation is questionable, especially for plots with significant differences in tree crown sizes. Secondly, estimating DBH through H and R and then using an allometric growth equation introduces additional procedural errors, leading to larger cumulative errors. In contrast, high-resolution image-based single-tree segmentation has advantages over solely using point cloud segmentation, and combining the two methods results in more accurate segmentation [45]. Using these more accurate parameters directly in the equation yields more precise calculations.

### 5.2 Applicability of the method based on canopy radius

Existing spaceborne LiDAR systems such as GEDI and ICESat-2 provide almost global coverage, uniform distribution, and high-density ground sampling data, facilitating the retrieval of canopy height information [25]. Fayad et al. evaluated their ability to detect canopy tops and their penetration capability in dense vegetation areas, demonstrating the potential of GEDI in forest ecosystems [46]. Although spaceborne LiDAR systems like GEDI cannot provide precise single-tree heights, they generally cover homogeneous areas where tree growth is similar. For sparse forests, the number of trees can be clearly counted on the sub-meter scale of satellite image. Combining spaceborne LiDAR data with crown radius information and utilizing the robust allometric growth models identified in this study, not only can be applied to large-scale areas but also greatly improve estimation accuracy. In future efforts, integrating images with improved spatial and spectral resolutions, along with crown width parameters and spaceborne LiDAR, holds promise for advancing forest AGB estimation on regional and global scales. In the future, it may be possible to estimate individual tree above-ground biomass (AGB) without relying on TLS and ALS by extracting the crown radius from remote sensing imagery and combining it with tree height obtained from satellite data, which could replace the use of diameter at breast height (DBH). For dense forests, where it is challenging to obtain accurate crown radius measurements, the models developed in this study are only applicable to trees that are well-suited for individual tree segmentation.

Nonetheless, by directly employing the conventional allometric growth equations and their coefficients outlined in the protocol, this study may not achieve precise estimates of single-tree above ground biomass (AGB). It could be beneficial to attempt determining coefficients for H and DBH through local experiments instead of directly adopting empirical values.

## 6. Conclusions

This study employed high-resolution data collected from drones to gather information on tree height and crown radius, thereby developing allometric growth models for the estimation of single-tree above ground biomass. Nine different allometric growth models were fitted for coniferous forests of varying heights, broadleaf forests, and mixed coniferous and broadleaf forests, respectively. Models with stronger applicability were selected for global-scale forest tree AGB estimation. The primary findings of this study are as follows: (1) Allometric growth models constructed with both tree height (H) and crown radius (R) perform better in estimating tree AGB compared to models constructed solely with "H" or "R". (2) The quadratic polynomial model constructed with "H+R" shows better fitting performance, particularly for mixed coniferous forests of varying heights. The quadratic polynomial model constructed based on tree height and crown radius demonstrates better performance in estimating individual tree above ground biomass. It demonstrates promising potential for substituting traditional allometric growth equations reliant on H and DBH. This approach fully utilizes canopy information, bypassing the difficulty of acquiring diameter at breast height in remote sensing estimation methods. It opens up possibilities for using high-resolution satellite data for global-scale forest AGB estimation.

In the future, we can explore the integration of crown information obtained from optical imagery with other remote sensing data, such as GEDI data, to conduct larger-scale and higher-accuracy AGB estimation studies. This approach aims to improve the accuracy of estimating individual tree above-ground biomass. The comprehensive use of different types of remote sensing information can provide a more complete perspective and more powerful tools for more precise biomass estimation.

## Supporting information

S1 File (XLSX)
